# A 2^nd^ Generation Linkage Map of *Heterobasidion annosum* s.l. Based on *In Silico* Anchoring of AFLP Markers

**DOI:** 10.1371/journal.pone.0048347

**Published:** 2012-11-05

**Authors:** Mårten Lind, Magriet van der Nest, Åke Olson, Mikael Brandström-Durling, Jan Stenlid

**Affiliations:** Department of Forest Mycology and Plant Pathology, Swedish University of Agricultural Sciences, Uppsala, Sweden; University of Queensland, Australia

## Abstract

In this study, we present a 2^nd^ generation genetic linkage map of a cross between the North American species *Heterobasidion irregulare* and *H. occidentale*, based on the alignment of the previously published 1^st^ generation map to the parental genomes. We anchored 216 of the original 308 AFLP markers to their respective restriction sites using an *in silico*-approach. The map resolution was improved by adding 146 sequence-tagged microsatellite markers and 39 sequenced gene markers. The new markers confirmed the positions of the anchored AFLP markers, fused the original 39 linkage groups together into 17, and fully expanded 12 of these to single groups covering entire chromosomes. Map coverage of the genome increased from 55.3% to 92.8%, with 96.3% of 430 markers collinearly aligned with the genome sequence. The anchored map also improved the *H. irregulare* assembly considerably. It identified several errors in scaffold arrangements and assisted in reducing the total number of major scaffolds from 18 to 15. This denser, more comprehensive map allowed sequence-based mapping of three intersterility loci and one mating type locus. This demonstrates the possibility to utilize an *in silico* procedure to convert anonymous markers into sequence-tagged ones, as well as the power of a sequence-anchored linkage map and its usefulness in the assembly of a whole genome sequence.

## Introduction

Genetic linkage maps have been used in genomic research for decades, both as a tool to identify candidate loci for phenotypic traits and as a way of studying chromosomal arrangement. Because of its potential for high yield of specific markers, a common fingerprinting method of choice has been amplified fragment length polymorphisms (AFLP) [1], in animals [2] and plants [2]–[3] as well as fungi [4]–[7]. As a consequence of recent improvements in and lowered costs for DNA sequencing technology, many organisms which previously have had their genome described with a genetic linkage map have since had their full genomes sequenced. These include a number of important fungi, such as *Pleurotus ostreatus* (http://genome.jgi.doe.gov/PleosPC15_2/PleosPC15_2.info.html), *Coprinopsis cinerea* [8], *Gibberella zeae* [9], *Mycosphaerella graminicola* [10], and the oomycete *Phytophthora infestans* [11]. A linkage map fully anchored to a whole genome sequence constitutes a very powerful tool, allowing immediate conversion of any mapped quantitative trait loci (QTL) to actual candidate genes. Without such a tool, the physical position of the QTLs will remain unknown, because the markers derived from random fingerprinting techniques like AFLP are anonymous and identification of candidate genes will require laborious map based cloning.

Anchoring and improving existing linkage maps assisted by genome sequence data has been done in various ways. In some cases the upgraded maps have been based only on new sequence-tagged markers without using the original map or sharing just a subset of the original markers to visualize the correlation between the maps [12]–[14]. Another approach has been to construct the new map complemented by markers derived from the known sequence [15]. Although these approaches can roughly link a map to its physical sequence, they don’t reveal the identity of every anonymous marker, which limits exploration of known QTLs. For organisms lacking known genome sequence, the process of converting anonymous markers into sequence-tagged sites has been even more cumbersome, usually involving repeated steps of selective AFLP with increasingly selective primers and manual excisions from polyacrylamide gels [16]–[17].

The root rot fungus *Heterobasidion annosum sensu lato* (s.l.) causes devastating damages to conifer forests all over the Northern Hemisphere. It is economically the world’s most important pathogen on conifers, causing yearly losses in Europe of at least €600 million [18]. The *Heterobasidion* species complex consists of phylogenetic distinctive, yet partially interfertile, species [19]–[20]. The interfertility phenomenon has been best described for the North American species *H. occidentale* and *H. irregulare*. It is controlled by at least five intersterility loci (S, P, V_1_, V_2_, V_3_) and compatible mating between individuals from the distinct species is possible only when both individuals in a pairing share a + allele for at least one of the intersterility loci [21]. However, the regular mating type system present in basidiomycetes is also present in *H. annosum* s.l. and is controlled by a bipolar mating system [22]–[23]. Albeit the system is controlled by a single MAT-A locus, there are probably more than a hundred mating type alleles in the species complex [24]–[25].

An AFLP markers-based linkage map of *Heterobasidion* species was originally published by Lind *et al* [26]. This 1^st^ generation map was based on a mapping population of 102 single spore isolates originating from a compatible mating [27] between a *H. occidentale* isolate (TC-122-12) and a *H. irregulare* isolate (TC-32-1) [23]. The map has been used to locate several traits of interest, such as growth rate [28], virulence [29], resistance to *Phlebiopsis gigantea* overgrowth [30] and various intraspecific interactions [31]. The interfertility between TC-32-1 and TC-122-12 was mediated through a common + allele for the V_3_ intersterility locus, while the S, P and V_2_ loci were all polymorphic between the parents. This allowed for the mapping of these three intersterility loci, but also of a mating type locus, as each progeny isolate will carry the MAT-A [22] allele from either one of the parents. Previous efforts to identify these loci [26] provided a strong position for the S locus, a weaker one for P and no position at all for V_2_ and MAT-A [26].

The aim of this study was to upgrade the *H. occidentale* x *H. irregulare* linkage map [26] into a sequence-based, 2^nd^ generation map covering the whole genome. We sought to achieve this by (i) *de novo* assemble the TC-122-12 parental strain genome and align it to the existing genome sequence of the TC-32-1 parental strain [23], (ii) anchor every AFLP marker of the original map to the genome sequences using an *in silico* approach; and (iii) to expand and fuse the anchored linkage groups into large groups covering whole individual chromosomes, by addition of new markers generated from the sequences of 146 SSRs and 39 known genes or ESTs. As a validation of the reliability of the final 2^nd^ generation map, we wanted to use it to position three intersterility loci and one mating type locus. Finally, the correlation between physical and genetic map enabled assessment of recombination rates across the intersterility and mating type loci.

## Materials and Methods

The parental strains TC-122-12 and TC-32-1 [32], the 102 progeny isolates used as a mapping population [27], the DNA extractions and the original linkage map [26] have been described elsewhere.

### Sequence Data and Genome Assembly

Genome sequence data for the *H. irregulare* parental strain TC-32-1 were obtained from the JGI genome project (http://genome.jgi-psf.org/Hetan2/Hetan2.home.html). Version 2 of the assembly comprised 33.6 MB distributed over 14 large (1.3–3.6 MB) and one small (8 KB) scaffolds [23].

*Heterobasidion occidentale* (TC-122-12) mycelium was cultivated and submitted to DNA extraction according to Lind *et al* [26]. Library preparations and sequencing took place at the SNP&SEQ Technology Platform of Uppsala University Hospital, using Solexa/Illumina short-read ultra-high throughput DNA sequencing technology (version 1.6) according to the manufacturers’ instructions. Paired-end sequences were obtained from libraries with an insert length of 400 bp and subsequently screened for poor quality using the SolexaQA software, version 1.13 [33]. The sequenced reads were then assembled with the Velvet assembler version 1.1.02 using parameters obtained from VelvetOptimiser version 2.2.0 [34]. Scaffolds larger than 1000 bp were aligned to the *H. irregulare* genome using the NUCmer function of MUMmer version 3.0. Based on this alignment *H. occidentale* scaffolds were assigned corresponding *H. irregulare* positions.

### Virtual AFLP

The principle of the virtual AFLP method, first described in 2003 [35], is to determine the genomic positions of AFLP markers obtained *in vitro* by an *in silico* simulation of the work of the restriction enzymes and the amplification steps. We used a DNA analysis software from AcaClone called pDRAW32 ver 1.1.110. Our markers to be anchored [26] were based on enzymatic cleavage with either *Pst*1-*Mse*1 or *Eco*R1-*Mse*1 pairs and amplified with two-nucleotide primer extensions. Thus, we scanned the TC-122-12 and TC-32-1 genomes for these pairs of restriction sites with the corresponding extensions, and selected candidates from pairs that flanked regions of the same size (in bp) as the sought markers.

### Identification of Microsatellites and Primer Design

Microsatellite markers were designed to confirm the positions of the anchored AFLP markers and to further improve the linkage map. The genome sequence of TC-32-1 was scanned for SSR motifs using the Magellan software [36] and one hundred and forty-six candidates were selected. Motifs of large core units (hexa or penta nucleotides) and high number of repeats (from 5× to 33×) were primarily chosen to increase the possibility of polymorphisms between the parents. Primers for each microsatellite were designed using the Primer3 web browser tool (http://bioinfo.ebc.ee/mprimer3/), with primer lengths varying between 18 and 22 bases, melting temperatures set between 59 and 61°C and a GC clamp of one nucleotide used. The primers were picked to amplify regions varying ideally between 100 and 500 bases in length, with a few exceptions (Table S1). The markers were then amplified from the mapping population according to the following procedure: 0.2 ng of template DNA to a final volume of 5 µl per reaction, containing 0.5 mM dNTP (Fermentas), 1.8 mM MgCl_2_, 1 µl Taq buffer (Fermentas), 0.5 U DreamTaq Green DNA polymerase (Fermentas) and 0.4 µM of each primer (TAG Copenhagen). The reactions were run for 35 cycles of denaturation (50 s, 95°C), annealing (50 s, 60°C), and extension (80 s, 72°C). The PCR products were in a few cases analyzed on an ABI Prism 310 Genetic Analyzer (PE Applied Biosystems) and the fragment lengths analyzed using the softwares GeneScan (PE Applied Biosystems) and Genotyper (PE Applied Biosystems). However, in most cases, the PCR products were analyzed by the Uppsala Genome Centre, using an ABI 3730 XL (PE Applied Biosystems).

### Segregation Analysis of Sequenced Genes

A set of 16 genes previously described as associated with fungal-fungal interactions in *Amylostereum areolatum*, *Neurospora crassa* and *H. irregulare* [37]–[40] was selected, along with 23 genes from an EST library [41]. Specific primers for all 39 genes were designed using Primer3. Alleles were distinguished in the parental genomes TC-122-12 and TC-32-1 using PCR-RFLP (restriction fragment length polymorphism) [42] analysis (50 cycles, denaturation (30 s, 95°C), annealing (30 s, 60°C), extension (30 s, 72°C)) and agarose gel electrophoresis. The genes were mapped by scoring allele segregation in the mapping population [27] using the EvaGreen dye (Biotium Inc., Hayward, CA, USA) together with post-PCR DNA High Resolution Melting curve analysis [43].

### Linkage Analysis

Segregation patterns for the 146 microsatellite markers and the 39 sequenced genes throughout the mapping population [27] were analyzed together with the existing set of 358 AFLP markers using the JoinMap 3.0 software [44]. The data set was coded as derived from haploid strains, originating from a diploid parent. Mapping groups were created of every cluster of markers joined together by a LOD score of 4 or higher. JoinMap suggested an internal marker order within each group. If the suggestion was in conflict with the physical position of the anchored markers, it was rejected. A manual suggestion was then created, with the markers arranged according to the anchored order. If this suggestion conflicted with the mapping data, the conflicting marker was moved within the suggested order, to a maximal extent of two markers away from its anchored positions (in one single case, three markers away). If the order was still not accepted, the conflicting marker was omitted. If the marker composition of a linkage group was supported by sequence based evidence, a LOD score of 2 was used as threshold for accepting the group. The sequence-anchored linkage map was visualized using MapChart 2.1 [45].

To compare the quality of sequence based and a non-sequence based genetic linkage map of the same genome, we estimated the genome coverage of the 1^st^ generation map [26] prior to improvement by new markers or adjusted marker orders. The base pair coverage of each anchored linkage group was calculated based on the largest possible physical distance between any two markers in a linkage group and compared to the coverage of the 2^nd^ generation map.

### Mapping Mating Type Genes and Intersterility Genes

The scoring and mapping procedure for the mating type locus and intersteriliy loci have been previously described [26]. Mapping data for intersterility loci P and V_2_ were obtained by crossing the mapping population with tester strains known to carry a + allele for either the P locus (strain Sä 16-4) or the V_2_ locus (strain TC-111-4). The previous attempt at this was incomplete, as just 39 and 28 of the 102 progeny isolates expressed fertility with the respective tester strain (51 expected) [26]. We made new crosses to obtain a complete set of observations. To estimate and analyze local recombination rates across the mating type and intersterility loci, KB/cM-rates were calculated continuously across every linkage groups using a sliding window of two markers, moving one marker at a time.

## Results

### Assembling the H. occidentale (TC-122-12) Genome Sequence

The *H. occidentale* strain TC-122-12 was sequenced with an Illumina Genome Analyzer, resulting in 61 million quality screened paired-end reads from a 400-bp insert library. The Velvet assembler *de novo*-assembled the reads into 19 848 contigs with an N50 of 34 641 bp, covering in total 28.5 MB (internal gaps excluded). The single largest contig was 217 732 bp, while another 2 625 contigs were at least 1000 bp large. These were subsequently aligned to the assembly of *H. irregulare* strain TC-32-1 [23] using NUCmer. NUCmer identified 10 834 matches between the assemblies of TC-122-12 and TC-32-1, ranging in length from 66 bases up to 42 942 bases. The matches ranged in similarity from 65.4% to 100% identical. Median match similarity was 89.1%.

### In Silico Anchoring AFLP Markers and Linkage Groups

**Figure 1:**
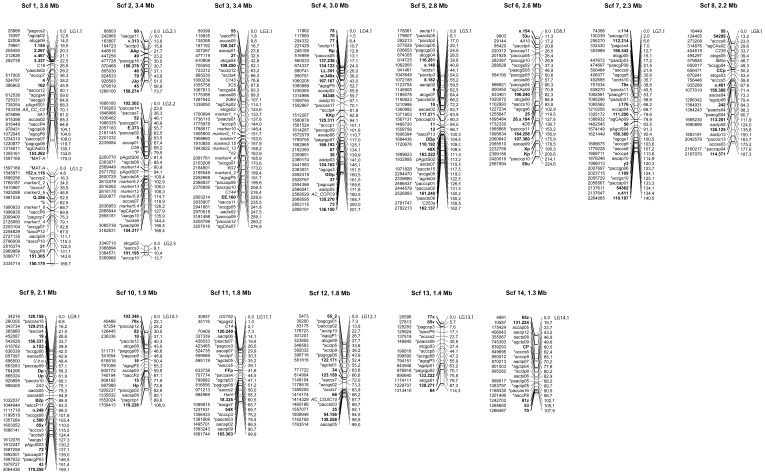
The 17 linkage groups of the 2^nd^ generation map of the *Heterobasidion annosum s.l.* genome. To the far left of each group bar is the position in basepairs on the *H. irregulare* or *H. occidentale* genome assembly. Next follow the marker names, where normal font indicates an AFLP marker or a mapped trait, **bold** stands for a microsatellite marker and *italic* names denotes a mapped gene of known sequence. To the right of the bar is the position in cM. Each group header tells the scaffold number associated with the group and its size in Mbp.

A virtual AFLP approach was used to identify the pairs of restriction sites flanking each AFLP marker of the 1^st^ generation linkage map [26]. To be considered as a candidate marker position, the distance between a pair of restriction sites had to be no more than 6 basepairs larger or smaller than the size given by the fragment analyzer (ABI Prism 310 Genetic Analyzer, PE Applied Biosystems) when the marker was originally scored. This frame was chosen to account for the potential misreads of the analyzing equipment and/or single nucleotide errors of the genome sequence. In the event that a tested marker had more than one putative genomic location, a closely linked marker was also tested, assuming that the markers would anchor to relatively proximate positions. Two linked markers rarely had more than one mutually putative position, and analyzing three linked markers was always enough to unambiguously locate them all. This was confirmed by microsatellite markers from the sequence covered by the anchored markers (see below and Figure 1).

One hundred and twenty-seven TC-32-1 AFLP marker positions from the original map (80% of all) were successfully anchored to the *H. irregulare* genome assembly by use of the virtual AFLP-approach. The NUCmer-mediated alignment of the *H. occidentale* and the *H. irregulare* assemblies made it possible to also convert 89 TC-122-12 AFLP markers positions (59% of all) to the corresponding positions on the *H. irregulare* reference genome. Anchoring these 216 marker positions also situated thirty-four of the 39 linkage groups of the map [26] to the genome. The five remaining groups were small; four contained 2 markers and one contained 3 markers.

### Creating a 2^nd^ Generation Linkage Map

One hundred and forty-six microsatellite markers segregated according to a 1∶1 ratio and were mapped with strong significance to different linkage groups. One hundred and forty-one of these had sequence support for their mapped positions, while five microsatellite markers mapped to positions without sequence support. Thirty-nine sequenced genes could also be mapped to existing linkage groups. BLASTing their sequences against the genome confirmed 35 of these mapped positions. The remaining four did not map to expected positions according to the BLAST search. Collectively, these 185 new markers significantly increased the quality of the map (Table 1 and 2, Figure 1), bringing the total number of markers up to 430, concentrating the number of groups from 39 down to 17, increasing markers per group from on average 7.9 to 25.3 and lowering the average distance between two neighboring markers from 7.3 to 5.8 cM, as compared between the 1^st^ and 2^nd^ generation maps. The rearrangement of markers due to their discovered physical positions, in addition to the mapped 146 microsatellite markers and 39 sequenced genes, discovered and omitted 68 falsely linked AFLP markers. Four hundred and fourteen of the 430 markers (96.3%) were assigned genetic positions (in cM) fully aligned to their physical position (in bp). The other 16 had to be assigned a skewed genetic position in order to fit the map, but markers distorted by a distance of more than two mapped markers away from the expected position was not accepted (except on one occasion) (Figure 1).

**Table 1:** Compared mapping data between 1^st^ and 2^nd^ *Heterobasidion annosum s.l.* linkage maps.

	2^nd^ gen	1^st^ gen
AFLP markers	241	308
SSR markers	146	–
Sequence markers	39	–
Traits	4	2
Total markers	430	310
Linkage groups	17	39
Avg markers per group	25.3	7.9
Mapped distance (cM)	2476	2252
cM/marker	5.8	7.3
KB/cM	12.6	5.8
Assembled size (KB)	33649	
Map coverage (KB)	31233	18603^a^
Map coverage (%)	92.8	55.3^a^

a The part of the assembly covered after anchoring the 1^st^ generation map, calculated on the largest distance between the anchored markers of a linkage group, disregarding order among markers.

After anchoring the 1^st^ generation map to the physical genome sequences of the parental strains, it covered 55.3% of the genome. After the addition of the 185 new markers, the entire 2^nd^ generation map covered 92.8%, which also increased the map-wide KB/cM level from 5.8 to 12.6 KB/cM (Table 2).

**Table 2:** Numeric data for each linkage group in the 2^nd^ generation linkage map for *Heterobasidion annosum s.l.*

Linkage group	Scaffold size b	Map covered (KB) c	Map covered (%)^c^	Size (cM)	KB/cM	Markers	AFLP	SSR	Gene	Traits	cM/marker
1.1	3592	1571	92.1	170	9.2	27	16	7	3	1 a	6.3
1.2		1738		169.7	10.2	20	9	5	4	2 a	8.5
2.1	3445	1219	79.7	73.7	16.5	13	3	10	0	0	5.7
2.2		1477		168.4	8.8	27	14	5	7	1	6.2
2.3		49		13.7	3.6	4	3	1	0	0	3.4
3.1	3434	3118	90.8	276.9	11.3	41	22	5	13	1	6.8
4.1	2988	2886	96.6	201.7	14.3	37	15	21	1	0	5.5
5.1	2842	2604	91.6	162.7	16	37	22	14	1	0	4.4
6.1	2573	2558	99.4	224.6	11.4	28	16	10	2	0	8
7.1	2304	2190	95.1	140.5	15.6	35	22	13	0	0	4
8.1	2239	2180	97.4	187.2	11.6	26	15	9	2	0	7.2
9.1	2115	2030	96.0	159.1	12.8	32	16	14	2	0	5
10.1	1875	1669	89.0	106.9	15.2	19	11	8	0	0	5.3
11.1	1817	1631	89.8	99.9	16.3	25	17	5	3	0	4
12.1	1764	1758	99.7	99.0	17.8	23	14	8	1	0	4.3
13.1	1386	1293	93.3	114.3	11.3	16	11	5	0	0	7.1
14.1	1267	1262	99.6	107.9	11.7	20	14	6	0	0	5.4
**Total**	**33641**	**31233**	**92.8**	**2476.3**	**12.6**	**430**	**240**	**146**	**39**	**5**	**5.7**

a Both groups contain mating type marker MAT-A.

b Size of the assembled scaffold or chromosome corresponding to the respective linkage group.

c Part of the assembled scaffold or chromosome covered by the respective linkage groupSupporting Information Legends.

### Locating Mating Type and Intersterility Loci

New data increased the observed number of progeny isolates capable of expressing fertility with the intersterility tester strain TC-111-4 (carrying the V_2_+ allele) from 28 to 42, and those expressing fertility with the tester Sä 16-4 (carrying the P + allele) from 39 to 54. This allowed us to use the 2^nd^ generation linkage map to locate the P-locus to scaffold 2, approximately between positions 2 239 084 and 2 250 770 bp in the *H. irregulare* genome (version 2, http://genome.jgi-psf.org/Hetan2/Hetan2.home.html). We were also able to position the V_2_-locus between 1 943 822 and 2 091 761 bp on scaffold 3. The genetic position of the previously mapped S-locus [26], was confirmed and anchored to scaffold 1 between positions 1 981 038 and 1 990 633 bp. The position of mating type locus MAT-A is known from the *H. irregulare* genome analysis to be located at 1 597 169 bp on scaffold 1 [23], in a poorly mapped area between the linkage groups 1.1 and 1.2. When mapped together with the markers from these groups, the mating type marker was positioned close to this locus in both groups, although the linkage data was insufficient to fuse the entire groups together.

The genome-wide recombination rate, calculated as the mean across all 17 linkage groups, was 12.6 KB/cM (Table 2). The mean for an individual linkage group (LG) (if disregarding the very small LG 2.3) spanned from 8.8 KB/cM (LG 2.2) to 17.8 (LG 12.1). Compared to this, recombination rates around the intersterility loci and mating type locus were in most cases elevated compared to the rest of genome (Figure 1). The putative position for intersterility locus S, on linkage group 1.2, had a recombination rate of 0.6 KB/cM, compared to a mean of 10.2 KB/cM for the linkage group as a whole. The suggested position for intersterility locus P has a recombination rate of 0.8 KB/cM, compared to the mean value of 8.8 for LG 2.2 in general. The mating type locus on LG 1.1, with a group mean of 9.2 KB/cM, has a local recombination rate of 6.8 KB/cM. The locus for intersterility locus V_2_ has a lower recombination rate than the rest of LG 3.1, 19.2 KB/cM compared to the group mean of 11.3 KB/cM. We also observed another local recombination hot spot close to the mapped position of V_2_ (8 cM away, between positions 1 908 660 and 1 910 855), with a recombination rate of 0.5 KB/cM. Elevated recombination rates of 1 KB/cM or less occurred between 29 neighbouring markers pairs throughout the linkage map. They were most frequent on LG 2.2 (5 hotspots) and did not occur at all on LGs 2.3, 8.1, 10.1 and 11.1. On LG 1.2, 2.2, and 3.1, containing the intersterility loci S, P and V_2_, we found 1, 5 and 3 hotspots, respectively (including the hotspots across the intersterility loci).

## Discussion

Using an *in silico* approach, 127 *H. irregulare* (TC-32-1) and 89 *H. occidentale* (TC-122-12) AFLP markers were anchored to the genome sequences of both species and incorporated into a 2^nd^ generation linkage map. This total of 240 is considerably less than the 308 AFLP markers present in the 1^st^ generation map, a decrease based on the exposure and omittance of falsely linked markers. Scoring errors and statistical ambiguities are known to cause a certain degree of false positive linkages during compilation of large sets of markers [46]. Data on physical positions is a powerful way to discern a false link from a correct one since the randomly occurring false linkages can easily be identified when a correct, sequence-based order of markers is enforced.

Although the 1^st^ generation map contained an equal amount of markers from each parent, more *H. irregulare* than *H. occidentale* markers remain in the 2^nd^ generation map (Table 2). The reason for this is that the *H. occidentale* genome by necessity was aligned using the *H. irregulare* genome as reference. The restriction sites of the 20 unanchored TC-122-12 markers probably exist among the 2 625 *H. occidentale* scaffolds, but some scaffolds might be too different from the *H. irregulare* genome to be aligned and positioned. Some of the missing markers might also be located in unassembled parts of the *H. occidentale* genome. Such are likely to exist since the 28.5 MB of the assembled *H. occidentale* genome represents only 85% of the 33.6 MB of the *H. irregulare* genome; a discrepancy probably accounted for by transposable elements, which were estimated to comprise 16.2% of the *H. irregulare* genome [23] and are probably also frequent in *H. occidentale*.

The 2^nd^ generation map covers 92.8% of the assembled *H. irregulare* sequence, compared to the 55.3% covered by the 1^st^ generation map. This massively improved coverage originates from the use of microsatellite markers to expand and merge formerly individual groups into larger ones. However, despite their physical closeness, linkage groups 1.1 and 1.2, and 2.1, 2.2 and 2.3, could not be joined. The areas between these groups contain a lot of transposable elements [23], which complicates the acquisition of unique microsatellite markers. If these unmapped areas contain recombinational hotspots or otherwise have high recombination rates, the groups would also be genetically unlinked.

The anchoring of AFLP markers revealed that four of the 39 linkage groups in the 1^st^ generation map [26] contained markers from two separate scaffolds. The other 35 solely consisted of markers from individual scaffolds, but the genetic position (in cM) of the markers never fully correlated to their physical positions (in bp). Incorrect marker orders impair the significance of QTL effects and complicate estimation of QTL sizes, which introduces uncertainty when identifying candidate genes. Our 2^nd^ generation map rectifies this problem by including only 16 markers with a genetic position in conflict with the physical position, and of these, only one is misplaced by a distance of more than two markers (three). The other 414 markers are mapped in concordance with sequence data which enables sharp and accurate QTL mapping. Furthermore, these results advice caution when interpreting QTL data from linkage maps without sequence-anchored markers, in terms of confidence in genome coverage, linkage group composition and marker order.

Of the 17 linkage groups (LG) remaining after the improvement of the map, twelve (LG 3.1–14.1) span almost the entire lengths of their respective scaffolds (Table 2), covering between 89% (LG 10.1) and 99.6% (LG 14.1) of the assembled sequence. None of the markers of these LGs showed any linkage to those of any other group in the 2^nd^ generation map, which suggests that the corresponding scaffolds represent individual chromosomes. This is also validated by the fact that six of the scaffolds have both their telomeric regions sequenced, and that the other six have one telomeric region sequenced [23]. Scaffold 1 may well also represent a chromosome, since (i) it has one sequenced telomeric region, (ii) LG 1.1 and 1.2 together cover 92.1% of its sequence, and (iii) the MAT-A locus links to the downstream end of LG 1.1 and upstream start of LG 1.2. Scaffold 2 is more obscure. Its three linkage groups only cover 79.7% of the sequence and it does not have any telomeric region sequenced. Since groups 2.1–2.3 does not link to any other groups, however, nothing contradicts that the scaffold represents a distinct chromosome. A fourteen chromosome arrangement was also suggested after the whole genome assembly of *H. irregulare* [23] and corresponds well to previous karyotyping data [47].

Anchoring this linkage map to sequence information was greatly beneficial for the assembling process of the *H. irregulare* genome. Version 1 of the assembly was constructed without consulting the map and consisted of 38 scaffolds, 18 of which were larger than 300 KB (http://genome.jgi-psf.org/Hetan1/Hetan1.home.html). In version 2 (http://genome.jgi-psf.org/Hetan2/Hetan2.home.html), map-based linkage information improved the assembly considerably. Scaffolds 16 and 12 of version 1 were joined into scaffold 9 of version 2, while scaffolds 11, 15 and 17 were joined into scaffold 4. Similarly, scaffolds 7 and 3 were split and joined into scaffolds 5 and 6. Map-based evidence thus decreased the total number of large scaffolds from 18 to 15. This highlights the usefulness of a high quality linkage map; it is not only a potent tool to target genomic regions governing phenotypic traits, but can also be used to fine-tune genome assemblies and increase our understanding of chromosomal arrangement.

Although the *in silico*-anchoring of AFLP markers is easiest with assemblies consisting of few, large scaffolds, it is potentially more beneficial for fragmented assemblies since it allows for joining of multiple scaffolds carrying linked markers. Although the number of candidate restriction sites could be more manageable in smaller genomes, the approach is also useful for larger ones; if the AFLP adapters and nucleotide extensions are specific enough to produce a unique marker *in vitro*, its original locus could also be determined *in silico*.

In the 1st generation map [26], the loci for MAT-A and V_2_ could not be positioned, but it was postulated that this was due to insufficient phenotype mapping data or to insufficient map coverage. Remedying these aspects enabled mapping of both loci, and the position for MAT-A (Figure 1) was confirmed by its physical position in the *H. irregulare* genome version 2 [23]. The molecular mechanism behind the intersterility concept is yet unknown, but since the occurrence of certain intersterility + alleles strictly follow the affinity for certain hosts [22], these traits might either be located physically close to each other or indeed be controlled by the same genes [21]. The putative loci are currently being examined for candidate genes, which will be investigated further using expression studies and gene deletions.

The most common approach to study genome-wide recombination rates is the so-called Marey mapping, i.e. comparing genomic and physical maps [48]. A simplified version of this was applied by running a sliding window across the scaffolds, comparing the distance in cM between pairwise neighbouring markers to their physical positions. Recombination frequencies measured in this way fluctuated a lot across the scaffolds, with in total 29 regional hotspots with a recombination rate below 1 KB/cM distributed over 13 of the 17 scaffolds. Interestingly, some of these hotspots coincided with the position for the intersterility loci S and P, and to a lesser extent with MAT-A. The mapped position for the V_2_ locus had no increased recombination rate, but just 8 cM away there was a position with a very similar recombination rate to S and P. Since only 42 of the assumed 51 existing isolates carrying the V_2_+ allele were identified, the current position is not necessary absolute and might in reality correlate to the neighbouring hot spot. The degree of recombination elevation were similar across all intersterility loci compared to the genome average of 12.6 KB/cM; for S, P and V_2_ respectively 0.6, 0.8 and 0.5 KB/cM, given that the local hot spot close to V_2_ is relevant. This is comparable to the increased recombination rate around the mating type locus measured in *Cryptococcus neoformans*, from 13.2 to 0.27 KB/cM [49].

The recombination rate in the region of MAT-A also increased (6.8 KB/cM), but not to the same extent as for the intersterility loci. This could be partially explained by the difficulties in mapping the mating type locus, physically situated in an area containing transposable elements. Affected recombination rates have been described previously for sex-determining regions of fungal genomes. In *Cryptococcus*, the increased recombination rates were accredited to flanking recombination hot spots on either side of the MAT-A locus, and it was suggested that this served to suppress recombination within the locus itself, as has also been described in *Neurospora tetrasperma* [50]. Hsueh *et al*. (2006) further argued the reason for this suppression to be the preservation of MAT as a single unit, as recombination within the locus could generate sterile or self-fertile progeny. The same argument could be made regarding the intersterility loci; if the increased recombination rate stems from flanking hot spots, the locus itself would be inherited as an intact entity. As individuals with disrupted intersterility loci would not carry any + alleles, they would invariably be sterile.

In this study, a *virtual* AFLP-approach was used to anchor AFLP markers from a previously published linkage map to the physical map. This provided the opportunity to determine the exact sequence for already identified and future QTLs. The reliability of this method was validated by microsatellite markers that confirmed the positions of the anchored AFLP markers. The genome coverage of the map was hence increased from 55.3% to 92.8% of the assembled sequence. This allowed the sharpened positioning of loci for intersterility and mating type, and the discovery that recombination rates were elevated around intersterility loci in particular. The described improvements to the linkage map also suggest that maps derived solely from random fingerprinting data might be fragmented, incomplete and assembled incorrectly, but of great use in genome assemblage following whole genome sequencing efforts.

## Supporting Information

Table S1 **Primer combinations for every microsatellite marker or mapped gene in the 2^nd^ generation linkage map of** ***Heterobasidion annosum s.l.***
(DOCX)
