# Supplementary material for: A 2nd Generation Linkage Map of Heterobasidion annosum s.l. Based on In Silico Anchoring of AFLP Markers
Source: PLoS One. 2012 Nov 5;7(11):e48347. doi: 10.1371/journal.pone.0048347 (PMC3489678; doi:10.1371/journal.pone.0048347)
Supplement: Table S1 — Primer combinations for every microsatellite marker or mapped gene in the 2nd generation linkage map of Heterobasidion annosum s.l. (DOCX) [file pone.0048347.s001.docx]

**Table S1.** Primer combinations for every microsatellite marker or mapped gene in the 2^nd^ generation linkage map of *Heterobasidion annosum s.l.*

| **Scaffold 1** | **Positions** | **Size** | **Sequence 5'-3' F/R** | |  |  |  |  |  |  |  |  |  |  |  |  |  |  |  |  |  |
| --- | --- | --- | --- | --- | --- | --- | --- | --- | --- | --- | --- | --- | --- | --- | --- | --- | --- | --- | --- | --- | --- |
|  | |  |  |  |  |  |  |  |  |  |  |  |  |  |  |  |  |  |  |  |  |
| 1.154 | 79681 | 158 | ACTCCCGTATTTAACCCATCC | | |  |  |  |  |  |  |  |  |  |  |  |  |  |  |  |  |
|  | 79839 |  | AAGCGAGACAATTGCGAGAC | | |  |  |  |  |  |  |  |  |  |  |  |  |  |  |  |  |
| 2.267 | 205450 | 267 | CAGGTACCTCTCCTCGAACG | | |  |  |  |  |  |  |  |  |  |  |  |  |  |  |  |  |
|  | 205717 |  | TGATCCTCAGGCCTCATTTC | | |  |  |  |  |  |  |  |  |  |  |  |  |  |  |  |  |
| x.467 | 212626 | 467 | ACCAAAATCAGCATCCTTGG | | |  |  |  |  |  |  |  |  |  |  |  |  |  |  |  |  |
|  | 213093 |  | GCTTGGAAGACTGGCTTGAC | | |  |  |  |  |  |  |  |  |  |  |  |  |  |  |  |  |
| 3.337 | 292738 | 343 | TACAAGCGGGGTCAGTAAGG | | |  |  |  |  |  |  |  |  |  |  |  |  |  |  |  |  |
|  | 293081 |  | GGTGCATATCGGGAAGTGAG | | |  |  |  |  |  |  |  |  |  |  |  |  |  |  |  |  |
| C16 | No position on scaffold | | GCACCACCGAGCACATAAAAG | | |  |  |  |  |  |  |  |  |  |  |  |  |  |  |  |  |
|  |  |  | TGTATACACGATGCTACTTCCG | | |  |  |  |  |  |  |  |  |  |  |  |  |  |  |  |  |
| 4 | 524787 | 260 | TCGAGTAGTTGTGGCGTCTG | | |  |  |  |  |  |  |  |  |  |  |  |  |  |  |  |  |
|  | 525047 |  | CGATCATAACGACGATACGC | | |  |  |  |  |  |  |  |  |  |  |  |  |  |  |  |  |
| 162 | 386902 | 162 | CTGTGCAACAACCAACAAGG | | |  |  |  |  |  |  |  |  |  |  |  |  |  |  |  |  |
|  | 387064 |  | AATCCCGATCAAACATCGAC | | |  |  |  |  |  |  |  |  |  |  |  |  |  |  |  |  |
| AA_CC_C2 | 753339 | 273 | GATGAGTCCTGAGTAACCAT | | |  |  |  |  |  |  |  |  |  |  |  |  |  |  |  |  |
|  | 753612 |  | GACTGCGTACCAATTCAAG | | |  |  |  |  |  |  |  |  |  |  |  |  |  |  |  |  |
| 5A1 | 875896 | 366 | CCATCGACAGCAACAACAAAAAT | | | |  |  |  |  |  |  |  |  |  |  |  |  |  |  |  |
|  | 876262 |  | GCGGAGCTGAGAGGAGTGC | | |  |  |  |  |  |  |  |  |  |  |  |  |  |  |  |  |
| 153.262 | 1355161 | 262 | TACAGGCGGGTATCTGAAGG | | |  |  |  |  |  |  |  |  |  |  |  |  |  |  |  |  |
|  | 1355423 |  | CACGCTGTCTTTGCTGTGAG | | |  |  |  |  |  |  |  |  |  |  |  |  |  |  |  |  |
| 152.x.116 | 1541675 | 171 | GGCCTGAACTGATTCCAAAC | | |  |  |  |  |  |  |  |  |  |  |  |  |  |  |  |  |
|  | 1541846 |  | GCCTCCTCCACACATGTACC | | |  |  |  |  |  |  |  |  |  |  |  |  |  |  |  |  |
| Marker2.2 | 1768187 | 331 | CGCGTACTTTGACGCTAACA | | |  |  |  |  |  |  |  |  |  |  |  |  |  |  |  |  |
|  | 1768518 |  | CTGCTGATCTACCGGAGGAG | | |  |  |  |  |  |  |  |  |  |  |  |  |  |  |  |  |
| Marker2.6 | 1925288 | 161 | GCATCGGCAGAAACAAGAG | | |  |  |  |  |  |  |  |  |  |  |  |  |  |  |  |  |
|  | 1925449 |  | TGTGTAGTTGCTGTAGGTCAAGC | | | |  |  |  |  |  |  |  |  |  |  |  |  |  |  |  |
| Q.356 | 1981038 | 361 | CCAATCCACTTCGATGCAC | | |  |  |  |  |  |  |  |  |  |  |  |  |  |  |  |  |
|  | 1981399 |  | GAAGGCCTTTGAAAATGACG | | |  |  |  |  |  |  |  |  |  |  |  |  |  |  |  |  |
| Marker1.4 | 2128180 | 270 | GAGAAGGCCACCAAGAAGAA | | |  |  |  |  |  |  |  |  |  |  |  |  |  |  |  |  |
|  | 2128450 |  | TGGTGAATCGTAAGGCAACA | | |  |  |  |  |  |  |  |  |  |  |  |  |  |  |  |  |
| 21 | 2815588 | 435 | GACACAGGATTCGCCTTTTC | | |  |  |  |  |  |  |  |  |  |  |  |  |  |  |  |  |
|  | 2816023 |  | GCAGCCGCTCTACTTACTGG | | |  |  |  |  |  |  |  |  |  |  |  |  |  |  |  |  |
| 151.305 | 3086717 | 305 | GCAATGTCTTCCCGAGAGTC | | |  |  |  |  |  |  |  |  |  |  |  |  |  |  |  |  |
|  | 3087022 |  | GATCACAGAACGAGCACACG | | |  |  |  |  |  |  |  |  |  |  |  |  |  |  |  |  |
| 150.179 | 3334714 | 178 | TCGCGTAGAGAGAGGAGAGG | | |  |  |  |  |  |  |  |  |  |  |  |  |  |  |  |  |
|  | 3334892 |  | CAGACTCCAGAGCCCAAAAG | | |  |  |  |  |  |  |  |  |  |  |  |  |  |  |  |  |
|  |  |  |  |  |  |  |  |  |  |  |  |  |  |  |  |  |  |  |  |  |  |
| **Scaffold 2** | |  |  |  |  |  |  |  |  |  |  |  |  |  |  |  |  |  |  |  |  |
| 80 | 68803 | 302 | CGAGTATGTGCGTCTGAAGG | | |  |  |  |  |  |  |  |  |  |  |  |  |  |  |  |  |
|  | 69105 |  | AATGTTGACCGACCCAACTC | | |  |  |  |  |  |  |  |  |  |  |  |  |  |  |  |  |
| v.313 | 163807 | 210 | TGCTGCCAACACATCTAACG | | |  |  |  |  |  |  |  |  |  |  |  |  |  |  |  |  |
|  | 164017 |  | ACGGCTGCTAGTGTTGCTG | | |  |  |  |  |  |  |  |  |  |  |  |  |  |  |  |  |
| AAp | 446616 | 203 | ACTCTGAATTTGCGCTCCTG | | |  |  |  |  |  |  |  |  |  |  |  |  |  |  |  |  |
|  | 446819 |  | GTAGAACTGCTCCGGTCCTG | | |  |  |  |  |  |  |  |  |  |  |  |  |  |  |  |  |
| 49 | 447256 | 161 | GCGGCACTTATCGACCTATC | | |  |  |  |  |  |  |  |  |  |  |  |  |  |  |  |  |
|  | 447417 |  | CGTATCGTCGTCGAGTCAAG | | |  |  |  |  |  |  |  |  |  |  |  |  |  |  |  |  |
| 156.378 | 570468 | 311 | GGCAGGAGCTGGTTCTAGTG | | |  |  |  |  |  |  |  |  |  |  |  |  |  |  |  |  |
|  | 570779 |  | TTGATTGCAGCCCTCTGTC | | |  |  |  |  |  |  |  |  |  |  |  |  |  |  |  |  |
| 46 | 805030 | 216 | TCGATGGTTTGCTGCTAATG | | |  |  |  |  |  |  |  |  |  |  |  |  |  |  |  |  |
|  | 805246 |  | GTGGGTCCCAATTTCTTGTG | | |  |  |  |  |  |  |  |  |  |  |  |  |  |  |  |  |
| 79 | 824533 | 239 | AATTGTTCGATGCGATCTCC | | |  |  |  |  |  |  |  |  |  |  |  |  |  |  |  |  |
|  | 824772 |  | ATCCCATTCACTTCGCACTC | | |  |  |  |  |  |  |  |  |  |  |  |  |  |  |  |  |
| JJp | 926583 | 293 | TCCTGTTCTCGAGGAACGTC | | |  |  |  |  |  |  |  |  |  |  |  |  |  |  |  |  |
|  | 926876 |  | TCCAACACATCTTGCTCTGG | | |  |  |  |  |  |  |  |  |  |  |  |  |  |  |  |  |
| 45 | 979519 | 223 | TCGTACACACACACGCACTC | | |  |  |  |  |  |  |  |  |  |  |  |  |  |  |  |  |
|  | 979742 |  | ATTTTGGGGGTGGAGAAAAC | | |  |  |  |  |  |  |  |  |  |  |  |  |  |  |  |  |
| 158.274 | 1288100 | 276 | TGCTTGCAAACACTCAGCTC | | |  |  |  |  |  |  |  |  |  |  |  |  |  |  |  |  |
|  | 1288376 |  | TGCTGCAGAAGACACTGAGG | | |  |  |  |  |  |  |  |  |  |  |  |  |  |  |  |  |
| 102.302 | 1686180 | 302 | GCCATTCAGACTGGAGGAAG | | |  |  |  |  |  |  |  |  |  |  |  |  |  |  |  |  |
|  | 1686482 |  | AACGTTGTTTGTGCAGGATG | | |  |  |  |  |  |  |  |  |  |  |  |  |  |  |  |  |
| 52 | 1936462 | 285 | CACGCTCCCGTATTTCTCTC | | |  |  |  |  |  |  |  |  |  |  |  |  |  |  |  |  |
|  | 1936747 |  | GGGATTATTTGGTCGTGCTG | | |  |  |  |  |  |  |  |  |  |  |  |  |  |  |  |  |
| E.373 | 2057183 | 376 | CAATAATGCTTCCCCTGCTC | | |  |  |  |  |  |  |  |  |  |  |  |  |  |  |  |  |
|  | 2057559 |  | AAAGCACAAGGGATGGACAC | | |  |  |  |  |  |  |  |  |  |  |  |  |  |  |  |  |
| 5 | 2201032 | 316 | CAGGAGAGTGCAGAGACGTG | | |  |  |  |  |  |  |  |  |  |  |  |  |  |  |  |  |
|  | 2201348 |  | CTTTTGGCCTCCTGAGTCTG | | |  |  |  |  |  |  |  |  |  |  |  |  |  |  |  |  |
| C59 | No position on scaffold | | CTTTATGGGACATTCTCGGG | | |  |  |  |  |  |  |  |  |  |  |  |  |  |  |  |  |
|  |  |  | ATCCAATGAATTTCCTAAGG | | |  |  |  |  |  |  |  |  |  |  |  |  |  |  |  |  |
| Marker14 | 2569449 | 351 | CTCCGAGGGAAGCTCTGGTA | | |  |  |  |  |  |  |  |  |  |  |  |  |  |  |  |  |
|  | 2569800 |  | AGGATGGACGACTTGGTCAC | | |  |  |  |  |  |  |  |  |  |  |  |  |  |  |  |  |
| Marker5.2 | 2612478 | 213 | GCGAAGTGCATGTGTGAAAC | | |  |  |  |  |  |  |  |  |  |  |  |  |  |  |  |  |
|  | 2612691 |  | AGGCAAGAGGAGATGAAGCA | | |  |  |  |  |  |  |  |  |  |  |  |  |  |  |  |  |
| Marker13 | 2616262 | 1753 | GACCTCGAGACCCTGATTGA | | |  |  |  |  |  |  |  |  |  |  |  |  |  |  |  |  |
|  | 2618015 |  | CTCAAGACAGCCTCCTTTGG | | |  |  |  |  |  |  |  |  |  |  |  |  |  |  |  |  |
| Marker12 | 2618176 | 1406 | AACTACGGCACCATCTGCTT | | |  |  |  |  |  |  |  |  |  |  |  |  |  |  |  |  |
|  | 2619582 |  | CCAATTTGGGACGTTCTTGT | | |  |  |  |  |  |  |  |  |  |  |  |  |  |  |  |  |
| Marker5.8 | 2620877 | 250 | CCCCGTTGTATGGAAAGCTA | | |  |  |  |  |  |  |  |  |  |  |  |  |  |  |  |  |
|  | 2621127 |  | CGGATCCAATCTTCATAGCC | | |  |  |  |  |  |  |  |  |  |  |  |  |  |  |  |  |
| Marker5.4 | 2636953 | 214 | TATCATTGAGCACGGAATCG | | |  |  |  |  |  |  |  |  |  |  |  |  |  |  |  |  |
|  | 2637167 |  | CTGTCCGCGCATATTTTCTT | | |  |  |  |  |  |  |  |  |  |  |  |  |  |  |  |  |
| 154.317 | 3162631 | 328 | CAAAGCATTTCGCATAGACG | | |  |  |  |  |  |  |  |  |  |  |  |  |  |  |  |  |
|  | 3162959 |  | GACAACTAGGGCACGGAGAC | | |  |  |  |  |  |  |  |  |  |  |  |  |  |  |  |  |
| 101.195 | 3384571 | 199 | AAGCTCCAGTTCCCGTTCTC | | |  |  |  |  |  |  |  |  |  |  |  |  |  |  |  |  |
|  | 3384770 |  | CGTTTATTATGCCCAGTACGC | | |  |  |  |  |  |  |  |  |  |  |  |  |  |  |  |  |
|  |  |  |  |  |  |  |  |  |  |  |  |  |  |  |  |  |  |  |  |  |  |
| **Scaffold 3** | |  |  |  |  |  |  |  |  |  |  |  |  |  |  |  |  |  |  |  |  |
| 55 | 89399 | 198 | CAGGGCCAGAATCAATTAGG | | |  |  |  |  |  |  |  |  |  |  |  |  |  |  |  |  |
|  | 89597 |  | TGTCAGCAGCAAACGAAAAG | | |  |  |  |  |  |  |  |  |  |  |  |  |  |  |  |  |
| 108.247 | 186470 | 247 | CCGGAAAAGAACACAGAACG | | |  |  |  |  |  |  |  |  |  |  |  |  |  |  |  |  |
|  | 186717 |  | TTTTTCTTTGGCGAGTGAGC | | |  |  |  |  |  |  |  |  |  |  |  |  |  |  |  |  |
| 27 | 477119 | 144 | GGATCCCACACATGTCCTTC | | |  |  |  |  |  |  |  |  |  |  |  |  |  |  |  |  |
|  | 477263 |  | ACGTGGGTCAGAAACAAAGC | | |  |  |  |  |  |  |  |  |  |  |  |  |  |  |  |  |
| 109.250 | 705550 | 252 | TGCAACAATTTCGAGGTGAC | | |  |  |  |  |  |  |  |  |  |  |  |  |  |  |  |  |
|  | 705802 |  | TGCTTCAGGATTTGCACTTG | | |  |  |  |  |  |  |  |  |  |  |  |  |  |  |  |  |
| C143 | 1000236 | 615 | GGGGGTAGAGAGGAGTAGAG | | |  |  |  |  |  |  |  |  |  |  |  |  |  |  |  |  |
|  | 1000815 |  | TAATGATGGGTATGCCTTGA | | |  |  |  |  |  |  |  |  |  |  |  |  |  |  |  |  |
| 3C6 | 1055758 | 317 | GATCTTTCCGTTCCCACCATTG | | |  |  |  |  |  |  |  |  |  |  |  |  |  |  |  |  |
|  | 1056075 |  | GCCGAGGCTGTCCCAAGTG | | |  |  |  |  |  |  |  |  |  |  |  |  |  |  |  |  |
| 20B4 | 1287542 | 415 | CACGGCAGACGCAAAGTAGAGC | | | |  |  |  |  |  |  |  |  |  |  |  |  |  |  |  |
|  | 1287957 |  | CCAGATGCCTAAGATTCGGACAAC | | | |  |  |  |  |  |  |  |  |  |  |  |  |  |  |  |
| Marker1.1 | 1700694 | 240 | TGTACTTCCACGTCGTCAGC | | |  |  |  |  |  |  |  |  |  |  |  |  |  |  |  |  |
|  | 1700934 |  | CCGGAGTAATCCGAGTTCAA | | |  |  |  |  |  |  |  |  |  |  |  |  |  |  |  |  |
| Marker10 | 1788517 | 1543 | CGAACCTGGGATTCAACATC | | |  |  |  |  |  |  |  |  |  |  |  |  |  |  |  |  |
|  | 1790060 |  | GTCTGCTCCTCTTCGTCACC | | |  |  |  |  |  |  |  |  |  |  |  |  |  |  |  |  |
| Marker2.11 | 1905900 | 163 | CATGCATGATGGCTACTCTGA | | |  |  |  |  |  |  |  |  |  |  |  |  |  |  |  |  |
|  | 1906063 |  | ATCCGGAGAGTATGGAGCTG | | |  |  |  |  |  |  |  |  |  |  |  |  |  |  |  |  |
| Marker7 | 1908660 | 1228 | CACCTCCGACAAACTTCGAT | | |  |  |  |  |  |  |  |  |  |  |  |  |  |  |  |  |
|  | 1909888 |  | CCTGACTCGTCTGTGCGTAA | | |  |  |  |  |  |  |  |  |  |  |  |  |  |  |  |  |
| Marker2.15 | 1910855 | 185 | CTTCGCTTGATGTTCCTCGT | | |  |  |  |  |  |  |  |  |  |  |  |  |  |  |  |  |
|  | 1911040 |  | ATGGTGGTCCTGCGTAAATC | | |  |  |  |  |  |  |  |  |  |  |  |  |  |  |  |  |
| Marker3.13 | 1943822 | 278 | CTGGAGCAGCTGAATGTGAC | | |  |  |  |  |  |  |  |  |  |  |  |  |  |  |  |  |
|  | 1944100 |  | TCGCTGCTTTGCAGTACATC | | |  |  |  |  |  |  |  |  |  |  |  |  |  |  |  |  |
| Marker4.4 | 2091761 | 408 | CATCCGAGCACTTGATTCCT | | |  |  |  |  |  |  |  |  |  |  |  |  |  |  |  |  |
|  | 2092169 |  | GGTCCCATGAAAGGAGGACT | | |  |  |  |  |  |  |  |  |  |  |  |  |  |  |  |  |
| 6G2 | 2164901 | 312 | AAGGCCGTCTGCACCAAGTC | | |  |  |  |  |  |  |  |  |  |  |  |  |  |  |  |  |
|  | 2165213 |  | GGCCGGATTCCTGCGTATT | | |  |  |  |  |  |  |  |  |  |  |  |  |  |  |  |  |
| Marker4.2 | 2307312 | 272 | CAACAACGGGGAGTCTATGG | | |  |  |  |  |  |  |  |  |  |  |  |  |  |  |  |  |
|  | 2307584 |  | TGGTAACTTGGAAGCCCTTG | | |  |  |  |  |  |  |  |  |  |  |  |  |  |  |  |  |
| C144 | 2376899 | 1065 | CGATATTCGCGAGTGTCTGTAGC | | | |  |  |  |  |  |  |  |  |  |  |  |  |  |  |  |
|  | 2377964 |  | CCAGCCATCATCCCATCCATA | | |  |  |  |  |  |  |  |  |  |  |  |  |  |  |  |  |
| EE.160 | 2803214 | 204 | CCTTCTCCTTCTCACCTTTCG | | |  |  |  |  |  |  |  |  |  |  |  |  |  |  |  |  |
|  | 2803418 |  | CATGACGTGTCGATAAGGACTATG | | | |  |  |  |  |  |  |  |  |  |  |  |  |  |  |  |
|  |  |  |  |  |  |  |  |  |  |  |  |  |  |  |  |  |  |  |  |  |  |
| **Scaffold 4** | |  |  |  |  |  |  |  |  |  |  |  |  |  |  |  |  |  |  |  |  |
| 78 | 11862 | 136 | CGGCCCTTCGTATTTATCTG | | |  |  |  |  |  |  |  |  |  |  |  |  |  |  |  |  |
|  | 11998 |  | CAAACATGTGAGCAGTGCAG | | |  |  |  |  |  |  |  |  |  |  |  |  |  |  |  |  |
| 76 | 117860 | 266 | CTTTACACGCTCCGACACAC | | |  |  |  |  |  |  |  |  |  |  |  |  |  |  |  |  |
|  | 118126 |  | TGATGTCCCTCCTTGAATCC | | |  |  |  |  |  |  |  |  |  |  |  |  |  |  |  |  |
| 77 | 204332 | 248 | ACCTCTGTCCTCGTCCACAC | | |  |  |  |  |  |  |  |  |  |  |  |  |  |  |  |  |
|  | 204580 |  | CTCTTCCCAGCGTGTCTCTC | | |  |  |  |  |  |  |  |  |  |  |  |  |  |  |  |  |
| Rp | 248188 | 121 | CCCTAGCAGCATTGATCGTC | | |  |  |  |  |  |  |  |  |  |  |  |  |  |  |  |  |
|  | 248309 |  | GATGGAGATGTGGGTCGTG | | |  |  |  |  |  |  |  |  |  |  |  |  |  |  |  |  |
| 137.236 | 560033 | 236 | TCAACCCTCAAATTCACACG | | |  |  |  |  |  |  |  |  |  |  |  |  |  |  |  |  |
|  | 560269 |  | TTGACAGGCCTAGGACAGAAC | | |  |  |  |  |  |  |  |  |  |  |  |  |  |  |  |  |
| 134.123 | 674337 | 126 | CTCTCAGCCTGTCTCGCTATC | | |  |  |  |  |  |  |  |  |  |  |  |  |  |  |  |  |
|  | 674463 |  | CTGTTCCTCTGACCCGAGAG | | |  |  |  |  |  |  |  |  |  |  |  |  |  |  |  |  |
| LL.351 | 890741 | 350 | TCCAGCGCTGTTCAGTATCTC | | |  |  |  |  |  |  |  |  |  |  |  |  |  |  |  |  |
|  | 891091 |  | AGGGACTTCCTTTCTAGGCAAC | | |  |  |  |  |  |  |  |  |  |  |  |  |  |  |  |  |
| w.350x | 956781 | 345 | ACCAGATCGTCTCGTTGTCC | | |  |  |  |  |  |  |  |  |  |  |  |  |  |  |  |  |
|  | 957126 |  | GCGACAGGTACGCATTGAC | | |  |  |  |  |  |  |  |  |  |  |  |  |  |  |  |  |
| 167.167 | 1060205 | 167 | TACCTGCGCGTACATCAAAC | | |  |  |  |  |  |  |  |  |  |  |  |  |  |  |  |  |
|  | 1060372 |  | AACAGCACATTCAGCACTGG | | |  |  |  |  |  |  |  |  |  |  |  |  |  |  |  |  |
| 54348 | 1314986 | 348 | CTGTGCAACAACCAACAAGG | | |  |  |  |  |  |  |  |  |  |  |  |  |  |  |  |  |
|  | 1315334 |  | AATCCCGATCAAACATCGAC | | |  |  |  |  |  |  |  |  |  |  |  |  |  |  |  |  |
| KKp | 1512287 | 249 | CTCCTGGATCTTGGGGATG | | |  |  |  |  |  |  |  |  |  |  |  |  |  |  |  |  |
|  | 1512536 |  | AGCTCTATGGGTCCGTCCTC | | |  |  |  |  |  |  |  |  |  |  |  |  |  |  |  |  |
| 125.311 | 1580878 | 288 | GGAATTAGATTGGGGCTGTG | | |  |  |  |  |  |  |  |  |  |  |  |  |  |  |  |  |
|  | 1581166 |  | TCGAATAACCCTCCTCATGC | | |  |  |  |  |  |  |  |  |  |  |  |  |  |  |  |  |
| 166.193 | 1882965 | 193 | CGAACCGCTAGAGAAAGACG | | |  |  |  |  |  |  |  |  |  |  |  |  |  |  |  |  |
|  | 1883158 |  | CATGCCACAACAGGGATATG | | |  |  |  |  |  |  |  |  |  |  |  |  |  |  |  |  |
| 67 | 2182360 | 343 | GATGCTGTTGAAGCCGAAG | | |  |  |  |  |  |  |  |  |  |  |  |  |  |  |  |  |
|  | 2182703 |  | TATACGCCCGTGGACTTTTG | | |  |  |  |  |  |  |  |  |  |  |  |  |  |  |  |  |
| 36 | 2359651 | 302 | TTTGCACAGGCGTACACTTC | | |  |  |  |  |  |  |  |  |  |  |  |  |  |  |  |  |
|  | 2359953 |  | TGGATTTTCCGTGCTATTCC | | |  |  |  |  |  |  |  |  |  |  |  |  |  |  |  |  |
| 124.162 | 2441583 | 164 | ATACCATGCGATGCAAGGAC | | |  |  |  |  |  |  |  |  |  |  |  |  |  |  |  |  |
|  | 2441747 |  | CGAGCCTCCAATCTAACCAG | | |  |  |  |  |  |  |  |  |  |  |  |  |  |  |  |  |
| GGp | 2492318 | 107 | TAGCACACTGCCGCAATATC | | |  |  |  |  |  |  |  |  |  |  |  |  |  |  |  |  |
|  | 2492425 |  | TGTTCGACGTTGGAAACTTG | | |  |  |  |  |  |  |  |  |  |  |  |  |  |  |  |  |
| 37 | 2528767 | 157 | CTTCTGAGCATCCCGTCATC | | |  |  |  |  |  |  |  |  |  |  |  |  |  |  |  |  |
|  | 2528924 |  | TACACGCTTCAAGAGCAAGC | | |  |  |  |  |  |  |  |  |  |  |  |  |  |  |  |  |
| AC_CCPC9 | 2568529 | 261 | ATCTGCTTATTGAACGAGGT | | |  |  |  |  |  |  |  |  |  |  |  |  |  |  |  |  |
|  | 2568790 |  | GTCCTCTAAGTGCACCATTG | | |  |  |  |  |  |  |  |  |  |  |  |  |  |  |  |  |
| 135.270 | 2668595 | 271 | AGCAAATCCACAGCCTTGTC | | |  |  |  |  |  |  |  |  |  |  |  |  |  |  |  |  |
|  | 2668866 |  | AGGTCGTGACAAATCGTTCC | | |  |  |  |  |  |  |  |  |  |  |  |  |  |  |  |  |
| 73 | 2853115 | 130 | TGAAGATCGTGCAGATGAATG | | |  |  |  |  |  |  |  |  |  |  |  |  |  |  |  |  |
|  | 2853245 |  | TGCGCCGTAAATCTACAATG | | |  |  |  |  |  |  |  |  |  |  |  |  |  |  |  |  |
| 136.150 | 2898191 | 150 | ACGACACAAATCCTGCTTCC | | |  |  |  |  |  |  |  |  |  |  |  |  |  |  |  |  |
|  | 2898341 |  | CGAGGTCGGACGAACTAAAG | | |  |  |  |  |  |  |  |  |  |  |  |  |  |  |  |  |
|  |  |  |  |  |  |  |  |  |  |  |  |  |  |  |  |  |  |  |  |  |  |
| **Scaffold 5** | |  |  |  |  |  |  |  |  |  |  |  |  |  |  |  |  |  |  |  |  |
| 116.281 | 914725 | 280 | CCCGCCTTTTCCTGTTATC | | |  |  |  |  |  |  |  |  |  |  |  |  |  |  |  |  |
|  | 915005 |  | CACGTTCAAAGATCGATTGC | | |  |  |  |  |  |  |  |  |  |  |  |  |  |  |  |  |
| s.148 | 1062955 | 189 | CTTTTCGATCTTGCGGACTC | | |  |  |  |  |  |  |  |  |  |  |  |  |  |  |  |  |
|  | 1063144 |  | TCAATTAGTGGAGGGCAAGG | | |  |  |  |  |  |  |  |  |  |  |  |  |  |  |  |  |
| 8.182 | 1072188 | 172 | ACTGCTCAACCCACTCCATC | | |  |  |  |  |  |  |  |  |  |  |  |  |  |  |  |  |
|  | 1072360 |  | CTTGAATTTTGCGGAAGAGG | | |  |  |  |  |  |  |  |  |  |  |  |  |  |  |  |  |
| 9 | 1145905 | 360 | TTGGTCTGAAAGCCAAAACC | | |  |  |  |  |  |  |  |  |  |  |  |  |  |  |  |  |
|  | 1146265 |  | TTCTGTTCAAACGCATGCTC | | |  |  |  |  |  |  |  |  |  |  |  |  |  |  |  |  |
| 10 | 1210986 | 167 | CATAGGCGTGGTAAGCAACC | | |  |  |  |  |  |  |  |  |  |  |  |  |  |  |  |  |
|  | 1211153 |  | GATGGGAGAGTGGTGGACTG | | |  |  |  |  |  |  |  |  |  |  |  |  |  |  |  |  |
| 117.271 | 1371903 | 271 | ACACAGTGCATCCGTCACAC | | |  |  |  |  |  |  |  |  |  |  |  |  |  |  |  |  |
|  | 1372174 |  | TGGGATCGAGGAGACCTTAC | | |  |  |  |  |  |  |  |  |  |  |  |  |  |  |  |  |
| 11 | 1468720 | 243 | GAACGCAACAAGAAGGAAGC | | |  |  |  |  |  |  |  |  |  |  |  |  |  |  |  |  |
|  | 1468963 |  | GCGTGTGAGTCGCATTATTTC | | |  |  |  |  |  |  |  |  |  |  |  |  |  |  |  |  |
| 12 | 1558756 | 174 | CCTGAGATGAGGTCCTGGAG | | |  |  |  |  |  |  |  |  |  |  |  |  |  |  |  |  |
|  | 1558930 |  | CTGCTGTGGTGTGGACAGAG | | |  |  |  |  |  |  |  |  |  |  |  |  |  |  |  |  |
| DDp | 1684436 | 169 | TGCAACTATCTGTCGCAAGC | | |  |  |  |  |  |  |  |  |  |  |  |  |  |  |  |  |
|  | 1684605 |  | AAGAAAAGCGTTTGCCGTAG | | |  |  |  |  |  |  |  |  |  |  |  |  |  |  |  |  |
| 118.192 | 1720676 | 191 | GGGCGAAATGATATGAAAGC | | |  |  |  |  |  |  |  |  |  |  |  |  |  |  |  |  |
|  | 1720867 |  | ATCCACCGACTTCAGATTGG | | |  |  |  |  |  |  |  |  |  |  |  |  |  |  |  |  |
| 40X | No position on scaffold | | CGCCTGAATACCCACCTG | | |  |  |  |  |  |  |  |  |  |  |  |  |  |  |  |  |
|  |  |  | AGCCCCAATGTGAGTCCAC | | |  |  |  |  |  |  |  |  |  |  |  |  |  |  |  |  |
| 163.222 | 1899623 | 222 | ACCAAAATCAGCATCCTTGG | | |  |  |  |  |  |  |  |  |  |  |  |  |  |  |  |  |
|  | 1899845 |  | GCTTGGAAGACTGGCTTGAC | | |  |  |  |  |  |  |  |  |  |  |  |  |  |  |  |  |
| 161.248 | 2526993 | 248 | CGACCACACTTGAGCAAAAC | | |  |  |  |  |  |  |  |  |  |  |  |  |  |  |  |  |
|  | 2527241 |  | ACCAATCAGACAAGGGCATC | | |  |  |  |  |  |  |  |  |  |  |  |  |  |  |  |  |
| C253a | 2781747 | 588 | CCGGGGGCTCACTCATCC | | |  |  |  |  |  |  |  |  |  |  |  |  |  |  |  |  |
|  | 2782335 |  | GTGCAATTGTAGCAGGGGTATCAGT | | | |  |  |  |  |  |  |  |  |  |  |  |  |  |  |  |
| 162.137 | 2782213 | 140 | TGCAGAGGTCATGAAATTCG | | |  |  |  |  |  |  |  |  |  |  |  |  |  |  |  |  |
|  | 2782353 |  | TTGAACACACCCGCAATATG | | |  |  |  |  |  |  |  |  |  |  |  |  |  |  |  |  |
|  |  |  |  |  |  |  |  |  |  |  |  |  |  |  |  |  |  |  |  |  |  |
| **Scaffold 6** | |  |  |  |  |  |  |  |  |  |  |  |  |  |  |  |  |  |  |  |  |
| n.154 | No position on scaffold | | CGAGGCGAAGACAACATACC | | |  |  |  |  |  |  |  |  |  |  |  |  |  |  |  |  |
|  |  |  | GTGAGCCGAAGAGATTGAGC | | |  |  |  |  |  |  |  |  |  |  |  |  |  |  |  |  |
| 53u | 9803 | 310 | AGTCGTGGTGGTCGATGAG | | |  |  |  |  |  |  |  |  |  |  |  |  |  |  |  |  |
|  | 10113 |  | TCTCTTGCGCTCTCCAGTTC | | |  |  |  |  |  |  |  |  |  |  |  |  |  |  |  |  |
| 4A10 | 29144 | 479 | AGCTGCTGAAATCTCTCGGTGTT | | |  |  |  |  |  |  |  |  |  |  |  |  |  |  |  |  |
|  | 29623 |  | GTGCCTCGCGAAGCCAG | | |  |  |  |  |  |  |  |  |  |  |  |  |  |  |  |  |
| 106.240 | 833461 | 240 | ATCTTTCACCCAGCGTTCTC | | |  |  |  |  |  |  |  |  |  |  |  |  |  |  |  |  |
|  | 833701 |  | TAGTGCCAGATTTGCGACAG | | |  |  |  |  |  |  |  |  |  |  |  |  |  |  |  |  |
| 25 | 1255647 | 153 | GCGTCGTGCTCGACTTTATC | | |  |  |  |  |  |  |  |  |  |  |  |  |  |  |  |  |
|  | 1255800 |  | GCACTCTGCTCGGCCTATAC | | |  |  |  |  |  |  |  |  |  |  |  |  |  |  |  |  |
| 26.x.184 | 1695484 | 292 | CCGCTGGAGAAATTCTGATG | | |  |  |  |  |  |  |  |  |  |  |  |  |  |  |  |  |
|  | 1695776 |  | TCTCAGCCAGCCATCAATC | | |  |  |  |  |  |  |  |  |  |  |  |  |  |  |  |  |
| 4D6 | 1732330 | - ^a^ | TGTCCAACCTGCCCCCAA | | |  |  |  |  |  |  |  |  |  |  |  |  |  |  |  |  |
|  | - ^a^ |  | AAACTTCACACCCCCACCCC ^a^ | | |  |  |  |  |  |  |  |  |  |  |  |  |  |  |  |  |
| 164.258 | 1995634 | 261 | CAAGCAAAACCCACACAATG |  |  |  |  |  |  |  |  |  |  |  |  |  |  |  |  |  |  |
|  | 1995895 |  | GCGTTCTGTCCTTTCTCCAG |  |  |  |  |  |  |  |  |  |  |  |  |  |  |  |  |  |  |
| 107.360 | 2060643 | 363 | TTCATACCTCGGTTCACACG | | |  |  |  |  |  |  |  |  |  |  |  |  |  |  |  |  |
|  | 2061006 |  | ACGACGCAAATTTCGATTTC | | |  |  |  |  |  |  |  |  |  |  |  |  |  |  |  |  |
| Xp | 2232759 | 203 | ACCCAGTTGGGACACAAGTC | | |  |  |  |  |  |  |  |  |  |  |  |  |  |  |  |  |
|  | 2232962 |  | GTGCTTCTGTCTCGCCATC | | |  |  |  |  |  |  |  |  |  |  |  |  |  |  |  |  |
| Kp | 2399156 | 164 | ACTCGCTGTGGTCAGTCCTC | | |  |  |  |  |  |  |  |  |  |  |  |  |  |  |  |  |
|  | 2399320 |  | CAGCGACGCACATGATACTC | | |  |  |  |  |  |  |  |  |  |  |  |  |  |  |  |  |
| 65u | 2567994 | 360 | AAGGTGCATTTTGGATCTGC | | |  |  |  |  |  |  |  |  |  |  |  |  |  |  |  |  |
|  | 2568354 |  | ATGGAGGGTATGCTCTCTGG | | |  |  |  |  |  |  |  |  |  |  |  |  |  |  |  |  |
|  |  |  |  |  |  |  |  |  |  |  |  |  |  |  |  |  |  |  |  |  |  |
| **Scaffold 7** | |  |  |  |  |  |  |  |  |  |  |  |  |  |  |  |  |  |  |  |  |
| r.114 | 74398 | 121 | CCCTAGCAGCATTGATCGTC | | |  |  |  |  |  |  |  |  |  |  |  |  |  |  |  |  |
|  | 74519 |  | GATGGAGATGTGGGTCGTG | | |  |  |  |  |  |  |  |  |  |  |  |  |  |  |  |  |
| 112.214 | 256270 | 214 | CCAAGCCCTAGCAATCAGAG | | |  |  |  |  |  |  |  |  |  |  |  |  |  |  |  |  |
|  | 256484 |  | GGCCAATGCAGAGCATAGAG | | |  |  |  |  |  |  |  |  |  |  |  |  |  |  |  |  |
| 156.342 | 353989 | 342 | GGCAGGAGCTGGTTCTAGTG | | |  |  |  |  |  |  |  |  |  |  |  |  |  |  |  |  |
|  | 354331 |  | TTGATTGCAGCCCTCTGTC | | |  |  |  |  |  |  |  |  |  |  |  |  |  |  |  |  |
| 78x | 751634 | 139 | CGGCCCTTCGTATTTATCTG | | |  |  |  |  |  |  |  |  |  |  |  |  |  |  |  |  |
|  | 751773 |  | CAAACATGTGAGCAGTGCAG | | |  |  |  |  |  |  |  |  |  |  |  |  |  |  |  |  |
| l.176 | 1065562 | 176 | CTGTGGTATCTGGCTGTGATTC | | |  |  |  |  |  |  |  |  |  |  |  |  |  |  |  |  |
|  | 1065738 |  | TCTCGAAACCGTCCAAGC | | |  |  |  |  |  |  |  |  |  |  |  |  |  |  |  |  |
| 111.200 | 1305172 | 229 | AGAGCTCGCTCCTGCTCTC | | |  |  |  |  |  |  |  |  |  |  |  |  |  |  |  |  |
|  | 1305401 |  | ATTCCCTTCTTGGGTCGTG | | |  |  |  |  |  |  |  |  |  |  |  |  |  |  |  |  |
| 56 | 1462545 | 325 | AAATATCCAGCCATGGTCAATC | | |  |  |  |  |  |  |  |  |  |  |  |  |  |  |  |  |
|  | 1462870 |  | CTCTCTGGCAAAGTGGTTTTG | | |  |  |  |  |  |  |  |  |  |  |  |  |  |  |  |  |
| 156.360 | 1621449 | 360 | GGCAGGAGCTGGTTCTAGTG | | |  |  |  |  |  |  |  |  |  |  |  |  |  |  |  |  |
|  | 1621809 |  | TTGATTGCAGCCCTCTGTC | | |  |  |  |  |  |  |  |  |  |  |  |  |  |  |  |  |
| y3 | 1996013 | 204 | GAGTGAAGCGAGCGAAAATC | | |  |  |  |  |  |  |  |  |  |  |  |  |  |  |  |  |
|  | 1996217 |  | CCCTTGCCTGAAGAAATCAC | | |  |  |  |  |  |  |  |  |  |  |  |  |  |  |  |  |
| r.109 | 2023715 | 121 | CCCTAGCAGCATTGATCGTC | | |  |  |  |  |  |  |  |  |  |  |  |  |  |  |  |  |
|  | 2023836 |  | GATGGAGATGTGGGTCGTG | | |  |  |  |  |  |  |  |  |  |  |  |  |  |  |  |  |
| x.411 | 2137647 | 411 | ACCAAAATCAGCATCCTTGG | | |  |  |  |  |  |  |  |  |  |  |  |  |  |  |  |  |
|  | 2138058 |  | GCTTGGAAGACTGGCTTGAC | | |  |  |  |  |  |  |  |  |  |  |  |  |  |  |  |  |
| 110.187 | 2264065 | 188 | AAATGCCATGAAAGCGTACC | | |  |  |  |  |  |  |  |  |  |  |  |  |  |  |  |  |
|  | 2264253 |  | TCCGACCAACCGATATTCTC | | |  |  |  |  |  |  |  |  |  |  |  |  |  |  |  |  |
|  |  |  |  |  |  |  |  |  |  |  |  |  |  |  |  |  |  |  |  |  |  |
| **Scaffold 8** | |  |  |  |  |  |  |  |  |  |  |  |  |  |  |  |  |  |  |  |  |
| 58 | 16449 | 179 | GTCGAGAGCGATGAGGAGAG | | |  |  |  |  |  |  |  |  |  |  |  |  |  |  |  |  |
|  | 16628 |  | TGACCATCTGAGCGTCAAAG | | |  |  |  |  |  |  |  |  |  |  |  |  |  |  |  |  |
| 54295 | 124405 | 295 | CTGTGCAACAACCAACAAGG | | |  |  |  |  |  |  |  |  |  |  |  |  |  |  |  |  |
|  | 124700 |  | AATCCCGATCAAACATCGAC | | |  |  |  |  |  |  |  |  |  |  |  |  |  |  |  |  |
| C236 | 404818 | 671 | CGATTTCGGCGGAGGACAC | | |  |  |  |  |  |  |  |  |  |  |  |  |  |  |  |  |
|  | 405489 |  | ATTGAGCCACGCGATAACTGC | | |  |  |  |  |  |  |  |  |  |  |  |  |  |  |  |  |
| 8B5a | 579824 | 336 | CGTCTTCCGTCCTCGCTCAGT | | |  |  |  |  |  |  |  |  |  |  |  |  |  |  |  |  |
|  | 580160 |  | ACCGCGCAGACGAAGGACA | | |  |  |  |  |  |  |  |  |  |  |  |  |  |  |  |  |
| CCp | 656471 | 159 | AGACGTGCATGGATCTACCC | | |  |  |  |  |  |  |  |  |  |  |  |  |  |  |  |  |
|  | 656630 |  | GTTGTCGTCGTCGTCCATC | | |  |  |  |  |  |  |  |  |  |  |  |  |  |  |  |  |
| x.461 | 935289 | 461 | ACCAAAATCAGCATCCTTGG | | |  |  |  |  |  |  |  |  |  |  |  |  |  |  |  |  |
|  | 935750 |  | GCTTGGAAGACTGGCTTGAC | | |  |  |  |  |  |  |  |  |  |  |  |  |  |  |  |  |
| 156.388 | 1077010 | 388 | GGCAGGAGCTGGTTCTAGTG | | |  |  |  |  |  |  |  |  |  |  |  |  |  |  |  |  |
|  | 1077398 |  | TTGATTGCAGCCCTCTGTC | | |  |  |  |  |  |  |  |  |  |  |  |  |  |  |  |  |
| 242 | 1364931 | 242 | AATCCCGATCAAACATCGAC | | |  |  |  |  |  |  |  |  |  |  |  |  |  |  |  |  |
|  | 1365173 |  | GGCAGGAGCTGGTTCTAGTG | | |  |  |  |  |  |  |  |  |  |  |  |  |  |  |  |  |
| 113.201 | 1668230 | 206 | AAAGAGAGAAGGGGGACGAG | | |  |  |  |  |  |  |  |  |  |  |  |  |  |  |  |  |
|  | 1668436 |  | GTTTAAGGTCAGTCGGTGTGG | | |  |  |  |  |  |  |  |  |  |  |  |  |  |  |  |  |
| 136.125 | No position on scaffold | | ACGACACAAATCCTGCTTCC | | |  |  |  |  |  |  |  |  |  |  |  |  |  |  |  |  |
|  |  |  | CGAGGTCGGACGAACTAAAG | | |  |  |  |  |  |  |  |  |  |  |  |  |  |  |  |  |
| 114.371 | 2197070 | 374 | GCTCCCCTCTAATTCATTTCG | | |  |  |  |  |  |  |  |  |  |  |  |  |  |  |  |  |
|  | 2197444 |  | GTAACGTATCCCCACCATGC | | |  |  |  |  |  |  |  |  |  |  |  |  |  |  |  |  |
|  |  |  |  |  |  |  |  |  |  |  |  |  |  |  |  |  |  |  |  |  |  |
| **Scaffold 9** | |  |  |  |  |  |  |  |  |  |  |  |  |  |  |  |  |  |  |  |  |
| 128.158 | 34216 | 158 | CAGACTTCGCTGTCGTTTTG | | |  |  |  |  |  |  |  |  |  |  |  |  |  |  |  |  |
|  | 34374 |  | GGGTACCACATTGGAACTGG | | |  |  |  |  |  |  |  |  |  |  |  |  |  |  |  |  |
| 129.213 | 343734 | 213 | GACGATGTCGATGTGTCACTG | | |  |  |  |  |  |  |  |  |  |  |  |  |  |  |  |  |
|  | 343947 |  | GAGGGGAGGGTAGCGTAAAG | | |  |  |  |  |  |  |  |  |  |  |  |  |  |  |  |  |
| 19 | 452087 | 379 | CCGCGAAGATAATGAAGAGG | | |  |  |  |  |  |  |  |  |  |  |  |  |  |  |  |  |
|  | 452466 |  | CAGCGGTCCTGGAATGTAAG | | |  |  |  |  |  |  |  |  |  |  |  |  |  |  |  |  |
| 156.337 | 542628 | 337 | GGCAGGAGCTGGTTCTAGTG | | |  |  |  |  |  |  |  |  |  |  |  |  |  |  |  |  |
|  | 542965 |  | TTGATTGCAGCCCTCTGTC | | |  |  |  |  |  |  |  |  |  |  |  |  |  |  |  |  |
| a.153 | 615762 | 160 | AGCGGAAGTACCTCATCGAC | | |  |  |  |  |  |  |  |  |  |  |  |  |  |  |  |  |
|  | 615922 |  | GCCGACAATAACCGAGAGTC | | |  |  |  |  |  |  |  |  |  |  |  |  |  |  |  |  |
| U.b.e | 696500 | 376 | CCTACATGCGCCTGTCTCGT | | |  |  |  |  |  |  |  |  |  |  |  |  |  |  |  |  |
|  | 696876 |  | TTGGGTGGTAAATCTTCGTCGTA | | |  |  |  |  |  |  |  |  |  |  |  |  |  |  |  |  |
| Dp | 764206 | 261 | TCAGAAGCAAGCACAGATGG | | |  |  |  |  |  |  |  |  |  |  |  |  |  |  |  |  |
|  | 764467 |  | CATCCTCAAGCAACGGATTC | | |  |  |  |  |  |  |  |  |  |  |  |  |  |  |  |  |
| Up | 868324 | 319 | CTTTCGTGCGCTGAAATAAG | | |  |  |  |  |  |  |  |  |  |  |  |  |  |  |  |  |
|  | 868643 |  | TAAAAAGCTGACACGCGAAC | | |  |  |  |  |  |  |  |  |  |  |  |  |  |  |  |  |
| 2A2 | 998405 | 421 | TCCTCGACCATCACCCTCTACCT | | |  |  |  |  |  |  |  |  |  |  |  |  |  |  |  |  |
|  | 998826 |  | TGGAGTATGCAGCGAGTTTGGA | | |  |  |  |  |  |  |  |  |  |  |  |  |  |  |  |  |
| B2p | 1032537 | 267 | ACGTACTCGGGTCTCATTGC | | |  |  |  |  |  |  |  |  |  |  |  |  |  |  |  |  |
|  | 1032804 |  | ATAGACCCCACACCCTCTCC | | |  |  |  |  |  |  |  |  |  |  |  |  |  |  |  |  |
| n.249 | 1111718 | 249 | CGAGGCGAAGACAACATACC | | |  |  |  |  |  |  |  |  |  |  |  |  |  |  |  |  |
|  | 1111967 |  | GTGAGCCGAAGAGATTGAGC | | |  |  |  |  |  |  |  |  |  |  |  |  |  |  |  |  |
| c.500 | 1357264 | 500 | TTAGCGCAGCAATCATAAGG | | |  |  |  |  |  |  |  |  |  |  |  |  |  |  |  |  |
|  | 1357764 |  | AGGGGATAATTTTCCAGATGC | | |  |  |  |  |  |  |  |  |  |  |  |  |  |  |  |  |
| 65x | 1603052 | 261 | AAGGTGCATTTTGGATCTGC | | |  |  |  |  |  |  |  |  |  |  |  |  |  |  |  |  |
|  | 1603313 |  | ATGGAGGGTATGCTCTCTGG | | |  |  |  |  |  |  |  |  |  |  |  |  |  |  |  |  |
| 72 | 1687258 | 432 | TGACTGACTGGCTGGATGAC | | |  |  |  |  |  |  |  |  |  |  |  |  |  |  |  |  |
|  | 1687690 |  | ATGTCTGCTGGCTGTACGTG | | |  |  |  |  |  |  |  |  |  |  |  |  |  |  |  |  |
| 43 | 1978727 | 136 | ATCCCGATCAGCATCAGC | | |  |  |  |  |  |  |  |  |  |  |  |  |  |  |  |  |
|  | 1978863 |  | AACAAAGACGTGCACAGACG | | |  |  |  |  |  |  |  |  |  |  |  |  |  |  |  |  |
| 170.258 | 2064436 | 258 | TGATGCTCACGCTAAACAGG | | |  |  |  |  |  |  |  |  |  |  |  |  |  |  |  |  |
|  | 2064694 |  | TGGTGTCGAAGCTTGATGAG | | |  |  |  |  |  |  |  |  |  |  |  |  |  |  |  |  |
|  |  |  |  |  |  |  |  |  |  |  |  |  |  |  |  |  |  |  |  |  |  |
| **Scaffold 10** | |  |  |  |  |  |  |  |  |  |  |  |  |  |  |  |  |  |  |  |  |
| 103.346 | No position on scaffold | | TTGGTATACTCGGCCTGGAC | | |  |  |  |  |  |  |  |  |  |  |  |  |  |  |  |  |
|  |  |  | TGTGCTCCAGAACAAAGACG | | |  |  |  |  |  |  |  |  |  |  |  |  |  |  |  |  |
| 70x | 40466 | 349 | TCCGCCCTACCTTAGTGTTG |  |  |  |  |  |  |  |  |  |  |  |  |  |  |  |  |  |  |
|  | 40815 |  | CACACTCAACTTTGCGAACG |  |  |  |  |  |  |  |  |  |  |  |  |  |  |  |  |  |  |
| 82 | 126445 | 168 | GCACGCGATGAGGTTAAATC |  |  |  |  |  |  |  |  |  |  |  |  |  |  |  |  |  |  |
|  | 126613 |  | CCACCGTCATCTTTCTCCAC | | |  |  |  |  |  |  |  |  |  |  |  |  |  |  |  |  |
| 18 | 238336 | 412 | TTGGGCTAGGGATTCATGTG | | |  |  |  |  |  |  |  |  |  |  |  |  |  |  |  |  |
|  | 238748 |  | TGCTGCCACTTATTTTCTGC | | |  |  |  |  |  |  |  |  |  |  |  |  |  |  |  |  |
| 16 | 616818 | 187 | CATCAAGAATTGGTGGCAAG | | |  |  |  |  |  |  |  |  |  |  |  |  |  |  |  |  |
|  | 617005 |  | CTGAAAGTACGCCCGAGTTC | | |  |  |  |  |  |  |  |  |  |  |  |  |  |  |  |  |
| 15 | 906182 | 289 | TGAATGGATTCGACATCAGC | | |  |  |  |  |  |  |  |  |  |  |  |  |  |  |  |  |
|  | 906471 |  | AGGAAGAAAGGGACCGTCTG | | |  |  |  |  |  |  |  |  |  |  |  |  |  |  |  |  |
| Hp | 987989 | 256 | ATTTAAGCTGGGCAGCACAC | | |  |  |  |  |  |  |  |  |  |  |  |  |  |  |  |  |
|  | 988245 |  | CACCTGTGGTACGTTCATCG | | |  |  |  |  |  |  |  |  |  |  |  |  |  |  |  |  |
| 119.226 | 1709413 | 239 | TCCGTCTGGCCAATTTCTAC | | |  |  |  |  |  |  |  |  |  |  |  |  |  |  |  |  |
|  | 1709652 |  | GATCAGCATACGAGGAGATCG | | |  |  |  |  |  |  |  |  |  |  |  |  |  |  |  |  |
|  |  |  |  |  |  |  |  |  |  |  |  |  |  |  |  |  |  |  |  |  |  |
| **Scaffold 11** | |  |  |  |  |  |  |  |  |  |  |  |  |  |  |  |  |  |  |  |  |
| GST82 | 30937 | 475 | CGGCATCGCGAAGGCGC | | |  |  |  |  |  |  |  |  |  |  |  |  |  |  |  |  |
|  | 31412 |  | TGCCGCCCAAGACAGGAG | | |  |  |  |  |  |  |  |  |  |  |  |  |  |  |  |  |
| C14 | No position on scaffold | | |  |  |  |  |  |  |  |  |  |  |  |  |  |  |  |  |  |  |
|  |  |  |  |  |  |  |  |  |  |  |  |  |  |  |  |  |  |  |  |  |  |
| 120.249 | 70409 | 278 | TCGGATCGCGTACTATGTTG | | |  |  |  |  |  |  |  |  |  |  |  |  |  |  |  |  |
|  | 70687 |  | CTCAAGCCCATTTGCTATCC | | |  |  |  |  |  |  |  |  |  |  |  |  |  |  |  |  |
| FFp | 632729 | 381 | TTGTGTCCAGATCAAATCCTG | | |  |  |  |  |  |  |  |  |  |  |  |  |  |  |  |  |
|  | 633110 |  | CCTTATAAGCGCAGGTAGGC | | |  |  |  |  |  |  |  |  |  |  |  |  |  |  |  |  |
| HahI | 994969 | 584 | GCTCCGGCCACCACCACGACTAT | | | |  |  |  |  |  |  |  |  |  |  |  |  |  |  |  |
|  | 995553 |  | AGACGGGCTGAACG | |  |  |  |  |  |  |  |  |  |  |  |  |  |  |  |  |  |
| 18.325 | No position on scaffold | | CGGTCTCGAGTCCTTGAATC | | |  |  |  |  |  |  |  |  |  |  |  |  |  |  |  |  |
|  |  |  | CTGCATCAAGGAGCCTCTTC | | |  |  |  |  |  |  |  |  |  |  |  |  |  |  |  |  |
| 54X | 1237531 | 258 | CTGTGCAACAACCAACAAGG | | |  |  |  |  |  |  |  |  |  |  |  |  |  |  |  |  |
|  | 1237789 |  | AATCCCGATCAAACATCGAC | | |  |  |  |  |  |  |  |  |  |  |  |  |  |  |  |  |
| 165.363 | 1661744 | 366 | GGATGGGAGCAGAGAAAATG | | |  |  |  |  |  |  |  |  |  |  |  |  |  |  |  |  |
|  | 1662110 |  | TTGCATTCTTTGTCGTGGAC | | |  |  |  |  |  |  |  |  |  |  |  |  |  |  |  |  |
|  |  |  |  |  |  |  |  |  |  |  |  |  |  |  |  |  |  |  |  |  |  |
| **Scaffold 12** | |  |  |  |  |  |  |  |  |  |  |  |  |  |  |  |  |  |  |  |  |
| 65_2 | 5473 | 375 | AAGGTGCATTTTGGATCTGC | | |  |  |  |  |  |  |  |  |  |  |  |  |  |  |  |  |
|  | 5848 |  | ATGGAGGGTATGCTCTCTGG | | |  |  |  |  |  |  |  |  |  |  |  |  |  |  |  |  |
| 122.171 | 581816 | 171 | TTTGCGAAGTTCAGGAGGAG | | |  |  |  |  |  |  |  |  |  |  |  |  |  |  |  |  |
|  | 581987 |  | CCTTCGTTCTTTCGTGACTTG | | |  |  |  |  |  |  |  |  |  |  |  |  |  |  |  |  |
| 34 | 771722 | 310 | GATGCCTTCCTTTCTCATGC | | |  |  |  |  |  |  |  |  |  |  |  |  |  |  |  |  |
|  | 772032 |  | GCGACATCCTCTCCAACTTC | | |  |  |  |  |  |  |  |  |  |  |  |  |  |  |  |  |
| 123.158 | 814904 | 158 | CGTCGGGAAGACGTATATGG | | |  |  |  |  |  |  |  |  |  |  |  |  |  |  |  |  |
|  | 815062 |  | CTCCTTTTGCTCCACTGCTC | | |  |  |  |  |  |  |  |  |  |  |  |  |  |  |  |  |
| 66 | 1414174 | 373 | ATTGCATGGGTGTAGCTTCC | | |  |  |  |  |  |  |  |  |  |  |  |  |  |  |  |  |
|  | 1414547 |  | GATACGATCCGCAAGCAATC | | |  |  |  |  |  |  |  |  |  |  |  |  |  |  |  |  |
| AC_CGSC10 | 1414329 | 239 | GACTGCGTACCAATTCACGCT | | |  |  |  |  |  |  |  |  |  |  |  |  |  |  |  |  |
|  | 1414568 |  | GATGAGTCCTGAGTAACGCC | | |  |  |  |  |  |  |  |  |  |  |  |  |  |  |  |  |
| 35 | 1557077 | 331 | CCATTCTTTTCGGACACCTC | | |  |  |  |  |  |  |  |  |  |  |  |  |  |  |  |  |
|  | 1557408 |  | GGCCACGAGTTAGAGTACCG | | |  |  |  |  |  |  |  |  |  |  |  |  |  |  |  |  |
| 54.156 | 1608948 | 156 | CTGTGCAACAACCAACAAGG | | |  |  |  |  |  |  |  |  |  |  |  |  |  |  |  |  |
|  | 1609104 |  | AATCCCGATCAAACATCGAC | | |  |  |  |  |  |  |  |  |  |  |  |  |  |  |  |  |
| 138.258 | 1742709 | 280 | CTTTCCTTCCCCTCTTCCAC | | |  |  |  |  |  |  |  |  |  |  |  |  |  |  |  |  |
|  | 1742989 |  | TAGCCGAAGCCAGCTATACG | | |  |  |  |  |  |  |  |  |  |  |  |  |  |  |  |  |
|  |  |  |  |  |  |  |  |  |  |  |  |  |  |  |  |  |  |  |  |  |  |
| **Scaffold 13** | |  |  |  |  |  |  |  |  |  |  |  |  |  |  |  |  |  |  |  |  |
| 77x | 20596 | 217 | ACCTCTGTCCTCGTCCACAC | | |  |  |  |  |  |  |  |  |  |  |  |  |  |  |  |  |
|  | 20813 |  | CTCTTCCCAGCGTGTCTCTC | | |  |  |  |  |  |  |  |  |  |  |  |  |  |  |  |  |
| 65v | 27813 | 365 | AAGGTGCATTTTGGATCTGC | | |  |  |  |  |  |  |  |  |  |  |  |  |  |  |  |  |
|  | 28178 |  | ATGGAGGGTATGCTCTCTGG | | |  |  |  |  |  |  |  |  |  |  |  |  |  |  |  |  |
| 133.222 | 890640 | 222 | CCACGAGTCGCTCTTGAA | | |  |  |  |  |  |  |  |  |  |  |  |  |  |  |  |  |
|  | 890862 |  | GCGCCGTGTTTGATAGAT | | |  |  |  |  |  |  |  |  |  |  |  |  |  |  |  |  |
| 130 | 1228707 | 271 | GCGATACGTCTAGCCTCCAG | | |  |  |  |  |  |  |  |  |  |  |  |  |  |  |  |  |
|  | 1228978 |  | CGTGAAGGACCTGGCTATTC | | |  |  |  |  |  |  |  |  |  |  |  |  |  |  |  |  |
|  |  |  |  |  |  |  |  |  |  |  |  |  |  |  |  |  |  |  |  |  |  |
| **Scaffold 14** | |  |  |  |  |  |  |  |  |  |  |  |  |  |  |  |  |  |  |  |  |
| 65z | 4891 | 362 | AAGGTGCATTTTGGATCTGC | | |  |  |  |  |  |  |  |  |  |  |  |  |  |  |  |  |
|  | 5253 |  | ATGGAGGGTATGCTCTCTGG | | |  |  |  |  |  |  |  |  |  |  |  |  |  |  |  |  |
| 131.224 | 10587 | 225 | CACAATGTACGCCTCTCTCG | | |  |  |  |  |  |  |  |  |  |  |  |  |  |  |  |  |
|  | 10812 |  | TTAATACCACGGGCAGAAGG | | |  |  |  |  |  |  |  |  |  |  |  |  |  |  |  |  |
| CP | 839293 | 210 | CGTCCAGAAATCCAGAATCC | | |  |  |  |  |  |  |  |  |  |  |  |  |  |  |  |  |
|  | 839503 |  | TCGTTCCTCGTTCTTCGTTC | | |  |  |  |  |  |  |  |  |  |  |  |  |  |  |  |  |
| 61z | 1242703 | 153 | GGTGTGGACGAGGTTAGAGG | | |  |  |  |  |  |  |  |  |  |  |  |  |  |  |  |  |
|  | 1242856 |  | CACACTCAACTTTGCGAACG | | |  |  |  |  |  |  |  |  |  |  |  |  |  |  |  |  |
| 53 | 1264650 | 315 | AGTCGTGGTGGTCGATGAG | | |  |  |  |  |  |  |  |  |  |  |  |  |  |  |  |  |
|  | 1264965 |  | TCTCTTGCGCTCTCCAGTTC | | |  |  |  |  |  |  |  |  |  |  |  |  |  |  |  |  |
| 70 | 1266487 | 225 | TTTCGGATATCGGTGGACTC | | |  |  |  |  |  |  |  |  |  |  |  |  |  |  |  |  |
|  | 1266712 |  | CGATCAATTGGATGGACCTC | | |  |  |  |  |  |  |  |  |  |  |  |  |  |  |  |  |

^a^ Reverse primer could not be found in the assembled sequence, likely due to assembly faults or gaps
